# Choice of Downscaled Climate Product Matters: Projections of Valley Fever Seasonality in a Warming Climate

**DOI:** 10.1029/2025GH001624

**Published:** 2025-12-31

**Authors:** Claire L. Schollaert, Simon Camponuri, Lisa Couper, Jennifer R. Head, Alexandra Heaney, Stefan Rahimi, Justin V. Remais, Miriam E. Marlier

**Affiliations:** ^1^ Department of Environmental Health Sciences UCLA Fielding School of Public Health Los Angeles CA USA; ^2^ Division of Environmental Health Sciences School of Public Health University of California, Berkeley Berkeley CA USA; ^3^ Department of Epidemiology School of Public Health University of Michigan Ann Arbor MI USA; ^4^ Institute for Global Change Biology University of Michigan Ann Arbor MI USA; ^5^ Herbert Wertheim School of Public Health and Human Longevity University of California San Diego San Diego CA USA; ^6^ Department of Atmospheric Science University of Wyoming Laramie WY USA

## Abstract

Downscaled climate projections provide valuable information needed to better understand the impacts of climate change on health outcomes and to inform adaptation and mitigation strategies at local to regional scales. Because downscaled climate products vary in their representations of fine‐scale spatiotemporal patterns, due to of multiple interacting factors, epidemiologic analyses need to consider how differences across downscaling approaches impact projections of health impacts into the future. We evaluate the projected seasonality of coccidioidomycosis in response to projected temperature and precipitation estimated using global climate models from CMIP6 included in California's Fifth Climate Change Assessment, downscaled using two approaches: (a) dynamical downscaling using the Weather Research and Forecasting model; and (b) hybrid statistical downscaling using the Localized Constructed Analogs approach. Our results indicate that by end of century, coccidioidomycosis transmission is projected to start earlier, end later, and last longer across the California endemic region; however, the magnitude of these changes varies by downscaling method. Specifically, LOCA2‐hybrid projected a season onset that is 4.2 weeks earlier and an end that is 4.1 weeks later than historical conditions, while the dynamical approach projected a 4 week earlier onset and a 3.8 week later end compared to the historical period. Overall, the LOCA2‐hybrid product estimates that the transmission season will last about 0.3 weeks longer than what is projected using dynamical downscaling by end of century. This analysis highlights the sensitivity of coccidioidomycosis seasonality projections to choice of downscaling product, underscoring the need to account for these differences in mitigation and adaptation planning.

## Plain Language Summary

Downscaled climate data are essential for projecting future local and regional climate‐related health impacts, but multiple downscaling approaches exist and each capture fine‐scale climate patterns differently. We examined how two common approaches for resolving global climate model data into local projections shape predictions of Valley fever seasonality in California. While both approaches suggest the transmission season will lengthen by the end of the century, they differ in how much change they predict. These differences matter because they can influence the timing of health warnings, resource allocation, and adaptation strategies. Our findings highlight the importance of considering climate data methods when using projections to guide climate and health research and planning.

## Key Points

- Dynamically and statistical downscaling produce different projections of future climate variables in central and southern California
- The California Valley fever season is projected to start earlier, end later, and thus last longer under future climate conditions
- The magnitude of these changes to future Valley fever seasonality in California are sensitive to choice of downscaled climate products

## 1 Introduction

Projections of the health impacts of climate change serve an important role in climate adaptation and public health preparedness. They can help anticipate future risks, guide adaptation efforts, and ensure that interventions are targeted to protect the most vulnerable populations (Hess et al., 2017). Assessing the magnitude and timing of health risks associated with climate change, along with the potential for adaptation and mitigation strategies to avert these risks, requires the joint analysis of spatiotemporally resolved climate, epidemiologic, and sociodemographic data (Conlon et al., 2016; Hess et al., 2017). Global climate models (GCMs) are used to simulate the dynamics of greenhouse gas and aerosol emissions, land use and land cover changes, and other socioeconomic and biophysical processes affecting the Earth's land surface, atmosphere, and oceans; however, their coarse spatial resolution (∼100 km^2^) limits their utility for impact assessments at local or regional scales (i.e., <15 km; Ekström et al., 2015; Jang & Kavvas, 2015; Walton et al., 2020).

These spatial constraints have led to the development of multiple downscaling methods designed to generate finer‐scale projections better suited for local impact assessments. Dynamical downscaling relies on regional climate models forced by boundary conditions from GCMs to simulate fine‐scale climate variability based on the governing equations (Bukovsky et al., 2021; Giorgi et al., 1994; Mearns et al., 2012; Rahimi et al., 2024a, 2024b; Rasmussen et al., 2023). While dynamical downscaling can be used to generate physically plausible climate trends, the process‐based modeling approach is computationally demanding, making it a less feasible option if downscaled data are sought across multiple emissions scenarios and realizations across many GCMs (Ekström et al., 2015; Walton et al., 2020). Statistical downscaling is an alternative and more widely used approach that relies on empirical relationships between GCM output and local observations to estimate finer‐scale climate variability. A variety of statistical approaches exist, including stochastic weather generators, regression methods, and weather typing approaches (Jang & Kavvas, 2015). Among the weather typing approaches, analog‐based methods, such as Localized Constructed Analogs (LOCA), rely on spatiotemporal analogs selected from the historical observational record to simulate future climate conditions (Pierce et al., 2014). While more computationally efficient, statistical downscaling is limited in its reliance on historical relationships, which may be invalid in the future (time stationarity). Finally, there are emerging hybrid methods that leverage the strengths of both statistical and dynamical downscaling, such as training LOCA using dynamically downscaled GCM outputs to improve statistically downscaled projections (Pierce et al., 2024; Walton et al., 2015).

Both dynamical and LOCA‐hybrid downscaling approaches have been used to generate high spatial resolution climate projections for California, as part of the state's Fifth Climate Change Assessment. These data products, as well as earlier versions, have been used to characterize the anticipated impacts of future climate on environmental exposures known to have adverse health impacts across California and the broader continental U.S, including extreme heat exposure (Ostro et al., 2011; Vahmani et al., 2019), projected urban ozone and fine particulate matter (PM_2.5_) exposures (Sun et al., 2015), drought and wildfire risk (McEvoy et al., 2020), and Valley fever (Gorris et al., 2021). While an increasing number of public health researchers are turning to the growing pool of publicly available downscaled climate data, it can be challenging to understand the effect of different downscaling techniques on projected health impacts. These nuances may be particularly important when projecting future impacts on risks that are dependent on intra‐annual changes in meteorological factors like temperature and precipitation, due to differences in how downscaling techniques capture these fine scale fluctuations in local climatology. Different downscaling techniques can also produce different climate trends across regions (Lafferty & Sriver, 2023), and this uncertainty may be propagated into downstream impacts assessments (Miller et al., 2025).

One such health risk associated with climate change that exhibits strong dependence on intra‐annual changes in meteorological factors is coccidioidomycosis, or Valley fever—an emerging fungal disease in the southwestern U.S. with approximately 20,000 cases reported each year. The disease is caused by inhaling spores of Coccidioides spp. fungi, which grow in soils and are aerosolized by soil disturbance or wind erosion. Because the fungi are endemic to arid and semi‐arid soils, soil conditions such as texture, salinity, and disturbance play an important role in shaping spore survival and release (Dobos et al., 2021). Coccidioidomycosis incidence is related to temperature, precipitation, and drought in California (Head et al., 2022): wet winters provide moisture for fungal growth, and a concomitant hot and dry summer and fall can create favorable conditions for spore aerosolization and dispersion, leading to increased incidence (Comrie, 2005; Head et al., 2022; Weaver & Kolivras, 2018). Coccidioidomycosis incidence generally follows a seasonal pattern, increasing in mid‐to late‐summer and declining during the winter (Comrie, 2005; Head et al., 2022; Heaney et al., 2024; Weaver & Kolivras, 2018). As climate change progresses in California, winters are expected to become wetter, while spring, summer, and fall seasons are expected to become hotter and drier. It follows that this sharpening of the wet season (i.e., lengthening of the dry seasons and delay of the wet season) may have important implications for coccidioidomycosis transmission (Swain et al., 2018). Indeed, prior work indicates that a shift from the 10th percentile to the 90th percentile of fall and spring precipitation could extend the coccidioidomycosis transmission season by 3.7 weeks (Camponuri, Head, et al., 2025; Camponuri, Heaney, et al., 2025).

With coccidioidomycosis incidence in California expected to change in response to shifts in intra‐annual temperature and precipitation patterns over the course of the century, it is imperative to understand how sensitive disease projections are to the resolution of the climate projections (i.e., choice of downscaling method) researchers use to project risk. Previous studies have evaluated the impact of downscaling methods on climate projections themselves, but few have assessed the sensitivity of downstream climate impacts to choice of downscaled climate product (Bhuvandas et al., 2014; Vogel et al., 2023). Studies that have evaluated the effects of downscaling methods on impact metrics have primarily focused on hydrological systems (Vogel et al., 2023) and ecological outcomes (Pourmokhtarian et al., 2016; Wang et al., 2017). One study did examine differences in downscaled temperature indices known to be associated with Salmonella spp. infections, but only considered statistical downscaling approaches and did not use those downscaled products to actually project future infections (Guentchev et al., 2016). Thus, the impact of downscaling method on variation and uncertainties in projected future climate‐related health burdens remains poorly understood.

**Figure 1:**
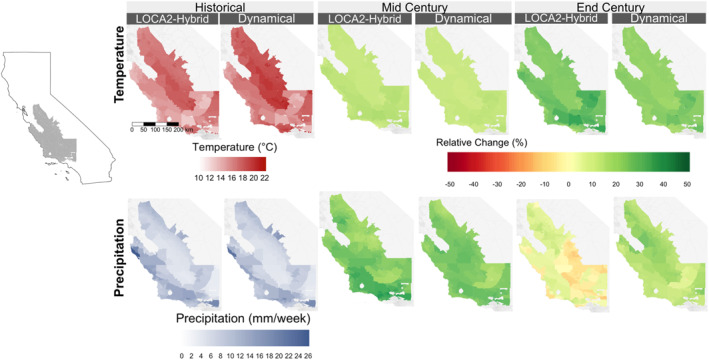
Study region census tract‐level average temperature (top row) and precipitation (bottom row) estimates across historical (1981–2010) and projected mid century (2035–2064) and end of century (2070–2099) periods derived using LOCA2‐hybrid and dynamical downscaling. The middle and right panels show relative change in average temperature and precipitation by mid and end of century relative to the historical period under each downscaling method.

**Figure 2:**
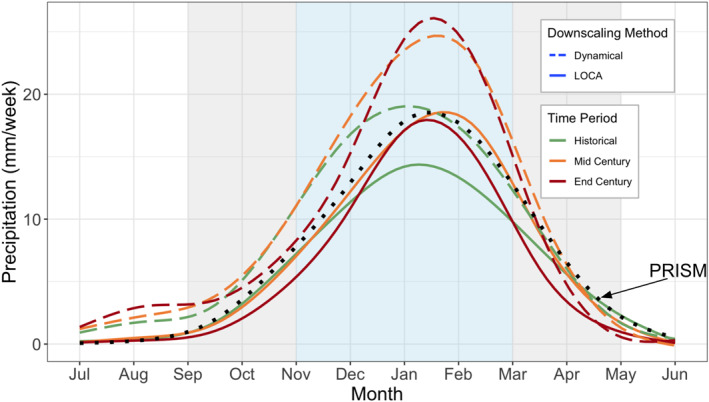
Average monthly precipitation by downscaling methods across the historical, mid century, and end of century time periods, smoothed using generalized additives models (GAM). The PRISM historical data are included as a reference in the dotted black line. The blue shading indicates the core wet season (November–March) and the gray shading indicates the shoulder seasons (September, October, April, May).

**Figure 3:**
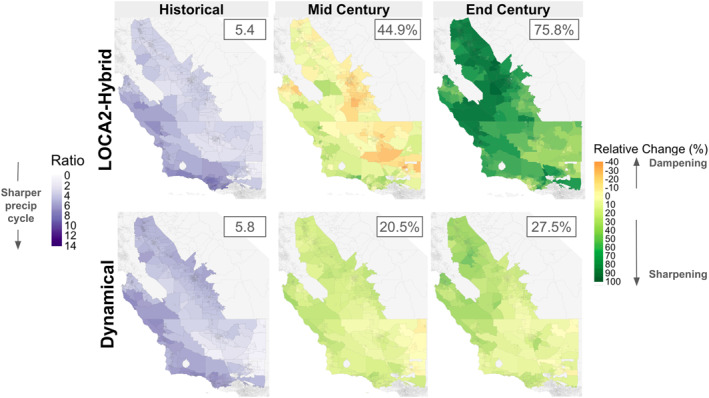
Average census tract‐level intra‐annual precipitation sharpness during the historical (left), mid century (middle), and end century (right) periods under both downscaling methods. Precipitation sharpness is expressed as the ratio of cumulative wet‐season (November‐March) precipitation to cumulative shoulder season (September, October, April, May) precipitation.

Prior comparisons of dynamically and statistically downscaled climate variables in California have identified key differences that may influence health impact projections. Walton et al. compared temperature and precipitation projections from the Center National de Recherches Météorologique Fifth Coupled Model (CNRM‐CM5), statistically downscaled to 6 km using LOCA and dynamically downscaled to 9 km using the Weather Research and Forecast (WRF) model and found that downscaled outputs differed more under future projections (2081–2100) than during historical baselines (1981–2000; Walton et al., 2020). Specifically, they estimated an average statewide absolute difference in springtime warming patterns between LOCA and WRF of 0.80°C by the end of the century, which is over three times the difference estimated during the historical period. Similarly, they estimated a statewide absolute difference in annual precipitation of 0.14 mm/day between the two methods, which is double the historical difference. These differences are particularly pronounced in the Sierra Nevada mountain range, where differences between methods increase to tenfold for temperature and fivefold for precipitation compared to historical differences, with WRF predicting much higher precipitation rates relative to LOCA in this region (Walton et al., 2020). These differences are driven by LOCA directly reflecting the climate signal from CNRM‐CM5, which are limited by their spatial resolution, while WRF develops its future climate scenarios by integrating fine‐scale dynamics from the boundaries of the modeling region. In other words, LOCA assumes that coarse resolution GCM model trends are preserved in high‐resolution temperature and precipitation fields, which is not the case with WRF. Walton et al. suggest that the dynamical approach provides a more accurate temperature output, while it remains unclear which downscaling method provides the more realistic precipitation prediction (Walton et al., 2020). As part of California's Fifth Climate Change Assessment, dynamically downscaled future GCM simulations were used to train LOCA to improve statistically downscaled climate predictions for the state (Pierce et al., 2024). This hybrid downscaling approach for California improves comparisons between projected temperature and precipitation estimates across statistical and dynamical downscaling approaches, though notable differences still persist (Figures 1, 2, 3), which may influence health impact projections.

Guidance on the selection of, and associated uncertainties in, downscaled climate data for health impacts projection is limited. Researchers must assess the validity of potential downscaled climate products, which can require an unreasonable level of literacy of the downscaling literature for researchers outside of climate science fields (Barsugli et al., 2013; Swart et al., 2017). While there is some discussion of statistical downscaling in the context of health impacts projection, uncertainties associated with choice of downscaling approach have generally gone unaddressed (Hess et al., 2017). Most discussion of downscaled data selection has focused on temporal resolution requirements (i.e., only dynamically downscaled data sets are available at sub daily time scales) and tradeoffs regarding number of GCMs and emissions scenarios available from dynamically versus statistically downscaled data sets (Kotamarthi et al., 2021; Pierce et al., 2024). Since these factors can come together to amplify uncertainties in projected impacts (e.g., changes in coccidioidomycosis incidence), public health researchers should evaluate the sensitivity of projected outcomes to choices made on downscaled data use.

Health impact projections require a set of interdisciplinary data inputs, including downscaled climate data. Here, we assess the sensitivity of future health impact projections to the choice of downscaled climate product. We explore how a subset of GCMs that have been downscaled for California using dynamical and hybrid statistical methods vary in their estimation of future local climate variables, with important consequences for projected seasonality of coccidioidomycosis risk in California (Camponuri, Head, et al., 2025; Camponuri, Heaney, et al., 2025; Comrie, 2005; Head et al., 2022; Heaney et al., 2024; Weaver & Kolivras, 2018). In particular, we project changes to the timing and duration of the coccidioidomycosis transmission season into the future using downscaled climate estimates, showing that choice of downscaled climate input data set does result in notable differences in these seasonality projections by end of century. Further, we provide a set of “key considerations” to facilitate the selection of an appropriate downscaling approach and data set for climate impact researchers.

## 2 Methods

### 2.1 Overview

We define the study region as the area of California endemic to coccidioidomycosis, based on prior work from Camponuri, Heaney, et al. (2025) which identified census tracts within areas with at least 500 total confirmed cases and average annual incidence rates >8 cases per 100,000 residents reported by the California Department of Public Health from 2000 to 2023. We compare temperature and precipitation projections for the study region (Figure 1) from three global climate models, downscaled both dynamically and statistically. We then use those downscaled climate variables to project future changes to coccidioidomycosis incidence based on a distributed‐lag Markov state‐transition model of seasonal coccidioidomycosis transmission (Camponuri, Heaney, et al., 2025). Coccidioidomycosis season onset, end, and duration were estimated for each census tract within the endemic region for a historical period (1981–2010), mid century (2035–2064), and end of century (2070–2099), and projections were assessed in relation to the choice of downscaling approach.

### 2.2 Climate Data

We used 4 km temperature and precipitation estimates from the Precipitation‐elevation Regressions on Independent Slopes Model (PRISM) as our historical baseline reference (Daly et al., 1994). PRISM was selected because it was used to establish the baseline relationship between coccidioidomycosis seasonality and meteorology by Camponuri, Head, et al. (2025), as described below. PRISM has been shown to perform particularly well in California relative to other commonly used gridded data sets (e.g., DayMet, Livneh, or ClimateNA) especially for precipitation (Stern et al., 2022). PRISM uses climate observations from several monitoring station networks across the United States and a variety of modeling approaches to interpolate between stations, generating a gridded climatological surface from 1981‐present. While technically a modeled data set, because it is rooted in station observations, we consider PRISM to reflect the observational record.

We used a set of three GCMs from the Coupled Model Intercomparison Project Phase 6 (CMIP6) that had been downscaled both dynamically with WRF and using the LOCA2‐hybrid statistical approach for California's Fifth Climate Change Assessment (Eyring et al., 2016). The suite of GCMs were selected based on the California Energy Commission's systematic evaluation of CMIP6 models for California (Krantz et al., 2021). This process ranked models using both process‐based metrics (e.g., representation of large‐scale circulation, ENSO, Santa Ana winds, blocking patterns, and extreme precipitation drivers) and local climate metrics (e.g., seasonal means and variability of temperature and precipitation over California). For this study, we prioritized models that performed well across both to ensure skill in reproducing observed California climate while also capturing the larger‐scale processes that drive regional variability. Our selection was based solely on performance in California and did not incorporate additional criteria such as region‐specific climate sensitivity or structural independence. From this evaluation, we selected three models that had been downscaled and bias‐corrected for CA using both methods and were available under Shared Socioeconomic Pathway SSP3‐7.0.: CNRM‐ESM2‐1, EC‐Earth3‐Veg, and FGOALS‐g3 (Döscher et al., 2022; Li et al., 2020; Séférian et al., 2019). CNRM‐ESM2‐1 is a fully coupled Earth system model with interactive carbon and aerosol cycles at a ∼1.4° resolution (Séférian et al., 2019). EC‐Earth3‐Veg is based on the ECMWF weather model and includes dynamic vegetation through LPJ‐GUESS, with a ∼0.7° resolution (Döscher et al., 2022). FGOALS‐g3 is the latest version of the Flexible Global Ocean–Atmosphere–Land System, with updated atmosphere and land components at ∼2.0° resolution (Li et al., 2020). SSP3.7.0 is one of five scenarios designed to reflect possible futures under different global climate policy options, and represents a medium to high future emissions scenario.

We used the 9 km bias corrected dynamically downscaled projections (O’Neill et al., 2016; Rahimi et al., 2024a, 2024b) along with the 3 km LOCA2‐hybrid downscaled projections (D. Pierce, 2023; Pierce et al., 2014). Dynamical downscaling uses regional climate models to simulate fine‐scale climate variability from GCM boundary conditions based on physical equations, while LOCA2‐Hybrid downscaling relies first on empirical relationships between GCM output and local observations, and then post hoc bias‐correction. While 3 km dynamically downscaled data do exist for California, their outputs were found to contain large biases in precipitation and temperature and, unlike LOCA2‐hybrid outputs, were not bias corrected in any way. With the 9 km data, Rahimi et al. reran identical WRF simulations in which the boundary conditions from the GCMs were bias corrected prior to downscaling (Rahimi et al., 2024a, 2024b). Compared to the original experiments, these new simulations demonstrated far superior skill in capturing the statistics of temperature and precipitation across the state, hence their use here. The LOCA2‐hybrid approach implements a more robust post‐downscaling quantile‐based seasonal bias correction to preserve the relationship between projections and observations (Pierce et al., 2024). For this analysis, we obtained both historical and future simulation for each GCM and downscaling method. Unlike the observed historical climate data sets, historical GCM simulations are trained and calibrated to historical observations but are driven by the physical processes within the climate models themselves. Minimum and maximum temperature were averaged and precipitation was summed across weeks and 2010 census tracts to match the spatial and temporal resolution of the original seasonality model (see below). PRISM temperature and precipitation data from 1981 to 2010 were averaged in the same manner.

### 2.3 Coccidioidomycosis Seasonality Model

The relationship between historical temperature, precipitation, and coccidioidomycosis season onset and end was established in a prior study, using a distributed‐lag Markov state‐transition model (Camponuri, Head, et al., 2025). The Camponuri et al. model used weekly census tract‐level data on incident coccidioidomycosis cases in California from 2000 to 2023 (*n* = 72,125) obtained with explicit permission from the California Department of Public Health's reportable disease surveillance system. Those case data are not used directly in the current analysis, only the model they were used to develop. The model treats each census tract's time series of cases as a continuous series of aseasonal and seasonal Markov states, where the instantaneous probability of transitioning from an aseasonal to seasonal state (i.e., season onset) and from a seasonal state to aseasonal state (i.e., season end) is modeled as a function of lagged temperature and precipitation. This previously developed framework captures both the timing and duration of seasonal transmission. In this study, we apply it with alternative downscaled climate data sets to generate weekly scale estimates of disease seasonality under different climate scenarios.

Similar regression‐based approaches have been applied to study climate impacts of Valley fever previously. For example, distributed‐lag nonlinear models and time‐series regression have been used to quantify the influence of precipitation, drought, and temperature on Valley fever incidence in California and the broader Southwest (Comrie, 2005; Gorris et al., 2018; Head et al., 2022). Wavelet analyses have been used to characterize the climate drivers of the seasonal dynamics of Valley fever in California (Heaney et al., 2024). State‐transition models have additionally been used in the context of influenza and other respiratory pathogens to capture seasonal forcing by temperature and humidity (Spiga et al., 2016).

### 2.4 Projecting Coccidioidomycosis Seasonality

To model projected changes in season onset and end under future climate conditions, we applied G‐computation substitution estimators derived in the original model (Camponuri, Heaney, et al., 2025; Robins, 1986), substituting average temperature and total weekly precipitation estimates from ensemble averages of the three GCMs for each downscaling method. To estimate season onset week for each census tract and year, we calculated the cumulative probability of season onset prospectively from the start of each transmission year (April 1–March 31), based on weekly projected temperature and precipitation data from each downscaled GCM ensemble. We then derived distributions of onset weeks for each census tract and year as the inverse (i.e., quantile function) of the cumulative onset probabilities. The average onset week was calculated from 10,000 Monte Carlo simulations of onset weeks from these distributions. Using the average estimated season onset week, we then calculated the cumulative probability of season end prospectively from season onset and calculated the average season end week for each census tract and year using the same approach. Season duration was calculated as the length of time from the estimated season onset to estimated season end. This process was carried out for the historical period, mid century, and end of century. We first generated season onset, end, and duration estimates for 1981–2010 using historical climate model simulations averaged across the three GCMs under both downscaling schemes as well as PRISM temperature and precipitation estimates as a comparative reference. We then generated projections of season onset, end, and duration for a mid century (2035–2064) and end of century (2070–2099) time periods using ensemble average temperature and precipitation estimates from the three GCMs for each downscaling method.

### 2.5 Comparisons Across PRISM and Downscaling Methods

Differences across downscaling methods can arise from both the methods themselves and the observational data sets used for training and bias correction. It is therefore important to appropriately define reference baselines when evaluating future projections. We first compared historical estimates of temperature, precipitation, and Valley fever seasonality derived from PRISM, the LOCA2‐hybrid, and the dynamically downscaled WRF output. These comparisons demonstrate how the downscaled products align with PRISM historically. PRISM is not used as an input to either downscaled product, but we include these comparisons to illustrate observational uncertainty and highlight potential differences among data sets that influence estimates of historical seasonality. We then compared future projections of temperature, precipitation, and Valley fever seasonality only to the historical baselines generated by each respective downscaling method (e.g., future LOCA2‐hybrid projections are evaluated relative to historical LOCA2‐hybrid estimates). This approach ensures internal consistency within each downscaling framework and reduces uncertainties due to differences in observational data sets when examining future projections.

## 3 Results

### 3.1 Differences in Precipitation and Temperature Estimates Across Downscaling Methods

Pearson correlations indicated minimal differences were observed in historical temperature and precipitation estimates between PRISM, GCM‐averaged LOCA2‐hybrid, and GCM‐averaged dynamically downscaled products. Weekly census tract‐level mean temperature estimates between 1981 and 2010 across the endemic study region showed high correlation across downscaling methods (LOCA2‐hybrid vs. Dynamical *R* = 0.99, PRISM vs. LOCA2‐hybrid *R* = 0.99, PRISM vs. Dynamical *R* = 0.98). Correlations remained high when broken down by season between downscaling methods (R range = 0.94–0.99) and when comparing LOCA2‐hybrid data to PRISM (R range = 0.97–0.98). When comparing dynamically downscaled weekly temperature to PRISM estimates at the seasonal level, correlation coefficients were slightly lower during spring, summer, and winter (*R* = 0.92–0.93), but still high during fall *R* = 0.97; Table S1 in Supporting Information S1).

Precipitation estimates between 1981 and 2010 across the endemic region also demonstrated strong correlation across downscaling methods, though the correlation between the downscaled GCMs and PRISM was slightly lower than that for temperature (LOCA2‐hybrid vs. Dynamical *R* = 0.96, PRISM vs. LOCA2‐hybrid *R* = 0.98, PRISM vs. Dynamical *R* = 0.95). Comparisons across methods become more variable when broken down by season, with correlation coefficients ranging from 0.85 to 0.98 across spring, fall, and winter, between the downscaled and reference methods. Correlations between precipitation estimates during the summer are much lower (LOCA2‐hybrid vs. Dynamical *R* = 0.33, PRISM vs. LOCA2‐hybrid *R* = 0.63, PRISM vs. Dynamical *R* = 0.00), likely due to minimal rainfall during the dry season (Table S1 in Supporting Information S1). Spatial comparisons of historical simulations under both downscaling methods relative to PRISM are in Figure S1 of Supporting Information S1.

Future projections for the endemic region showed consistent relative changes in annual temperature across downscaling methods and GCMs (Figure 1 and Figure S2 in Supporting Information S1). However, as compared to annual temperature projections, projections of annual precipitation had lower agreement across downscaling models, particularly by mid century, when dynamical downscaling predicts greater increases across the study region compared to LOCA2‐hybrid. By the end of the century, the southern half of the endemic region shows a slight decrease in average annual precipitation under the LOCA2‐Hybrid, that is not present in the dynamically downscaled estimates (Figure 1). Intra‐annual temperature changes were consistent across downscaling methods, while precipitation changes showed less agreement. Dynamical downscaling projected greater increases in winter precipitation, especially by the end of the century, as compared to the LOCA2‐hybrid average (Figure 3 and Figures S2, S3 in Supporting Information S1). These differences highlight how LOCA2‐hybrid assumes that coarse‐resolution trends from the GCMs are preserved in the future, while dynamically downscaling does not.

The wet season is projected to condense and become wetter throughout the century, with sharper transitions from wet to dry seasons—an effect seen under both downscaling schemes, but more distinct in LOCA2‐hybrid projections, particularly by the end of century (Figures 2 and 3). The LOCA2‐hybrid shows a greater sharpening, or increase in the proportion of total annual rainfall that falls within November ‐ March (84% during the historical period, 90% by end of century; Figure 2). While dynamical downscaling shows a similar pattern, it is less pronounced (86% during the historical period, 88% by end of century; Figure S4 in Supporting Information S1). We quantify “sharpness” as the ratio of cumulative core wet season (November‐March) precipitation to cumulative shoulder season precipitation (September‐October preceding and the April‐May following each wet season), with higher ratio values indicating sharper transitions between wet and dry seasons (or a greater precipitation “whiplash” effect; Swain et al., 2018). The LOCA2‐hybrid projects an average “sharpness” ratio of 7.9 by mid century (44.9% increase from historical conditions) and 9.3 by end of century (75.8% increase from historical conditions). This effect was less pronounced in dynamically downscaled projections, with an average “sharpness” ratio of 7.0 by mid century (20.5% increase from historical conditions) and 7.4 by end of century (27.5% increase from historical conditions), with some regions, like the southern coast, even experiencing a decrease in sharpness by the end of the century. These differences are due in part to preservation of historical trends in the LOCA2‐hybrid data, but also due to differences in bias correction approaches across downscaling methods. As noted previously, LOCA2‐hybrid is forced to match historical observations by quantile post‐downscaling, while the dynamically downscaled data undergo a less aggressive bias correction procedure pre‐downscaling.

### 3.2 Differences in Historical and Projected Coccidioidomycosis Seasonality Using Each Downscaling Method

**Figure 4:**
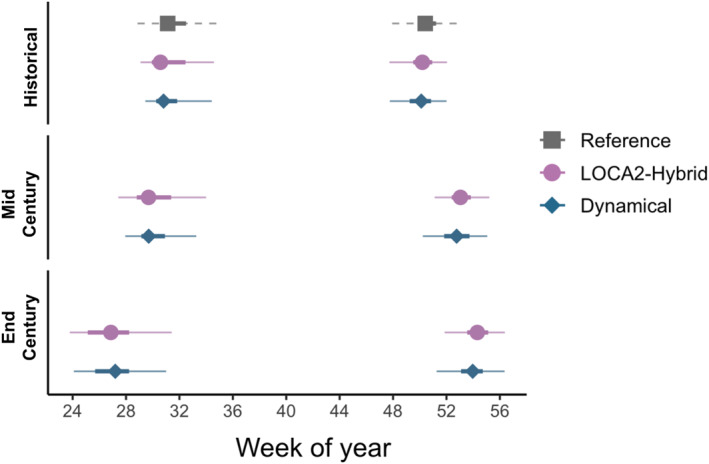
Median, IQR (thick lines), 5th, and 95th percentiles (thin lines) of season onset and end weeks over the course of the century estimated using temperature and precipitation projections from both downscaling methods and PRISM for the historical period. Bars represent the spread across census tracts and years within each time period.

Aggregated across the full study region, historical (1981–2010) coccidioidomycosis season onset and end week estimates show minimal differences between downscaled GCMs and PRISM. Transmission seasons typically begin in late July to early August and end by mid to late December across the endemic region. By mid century, season onset is projected to start earlier under both downscaling methods (averaging 1.1 weeks earlier under dynamical downscaling and 1 week earlier under the LOCA2‐hybrid), while season end is projected to be delayed into mid‐January under both methods (averaging 2.7 weeks later under dynamical downscaling and 2.9 weeks later under the LOCA2‐hybrid). By the end of the century, the season end is further delayed into late January, occurring an average of 3.8 weeks later under dynamical downscaling and 4.1 weeks later under the LOCA2‐hybrid. By end of century season, onset is projected to continue shift earlier, particularly in LOCA2‐hybrid estimates (2.9 weeks earlier on average) compared to dynamically downscaled projections (2.7 weeks earlier; Figures 4 and 6). The distribution of season onset and end week projections across GCMs is provided in Figure S5 of Supporting Information S1.

**Figure 5:**
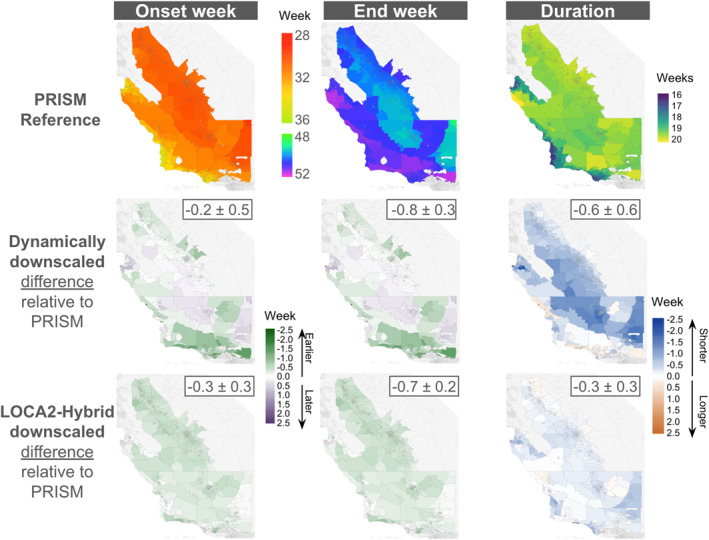
Census tract‐level season onset, end week, and duration estimates during the historical period, estimated using temperature and precipitation estimates from PRISM (top row). Differences in historical season onset, end week, and duration relative to the PRISM reference estimates using the dynamically downscaled historical simulations (middle row) and using the LOCA2‐hybrid historical simulations (bottom row). The boxes on the top right of each panel show average ± standard deviation difference relative to PRISM across the study region.

The spatial distribution of season onset, end week, and duration calculated using both downscaled GCMs and PRISM indicate that, historically, the transmission season commenced earlier in the Central Valley when compared with coastal regions. Season end was slightly delayed in mountainous coastal areas and the Sierra foothills. The spatial distribution of season duration followed a similar pattern, with longer transmission seasons in the Central Valley compared to coastal regions (Figure 5).

GCM historical simulations estimate season onset weeks on average 0.2 and 0.3 weeks earlier than PRISM under dynamical downscaling and the LOCA2‐hybrid, respectively. The season end week estimated using dynamically downscaled data averaged 0.8 weeks earlier than those estimated using PRISM data, while season end based on LOCA2‐hybrid averaged about 0.7 weeks earlier than PRISM. Under dynamical downscaling, these slightly earlier onset and end dates are primarily driven by the coastal mountains and Sierra foothills, whereas the Central Valley exhibits slightly later timing for both onset and end. Season durations were 0.6 weeks shorter, on average, when estimated using the dynamically downscaled data, compared to 0.3 weeks shorter when estimated using LOCA2‐hybrid (Figure 5).

**Figure 6:**
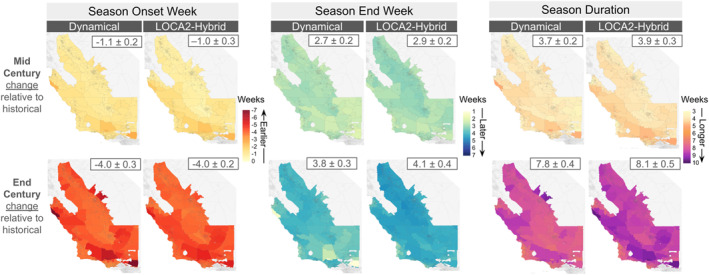
Change in future season onset, end week, and duration estimates by mid century (top) and end of century (bottom) relative to the historical simulations for each respective downscaling method. The box in the top right of each panel represents the mean ± standard deviation change across the full study region.

Our analyses projected an earlier onset and later end of the coccidioidomycosis transmission season in future decades, resulting in longer season durations. These changes were consistent across downscaling methods by mid century. By the end of century, however, spatial differences in projected seasonal trends across downscaling methods were more pronounced. Onset weeks were pushed earlier on the coast relative to the Central Valley when calculated using the dynamically downscaled data, while changes were more evenly distributed across the study area when projected using the LOCA2‐hybrid (Figure 6). Similarly, we saw larger delays in season end using the LOCA2‐hybrid data, relative to the dynamically downscaled data, that were more evenly distributed across the full study region. Changes in season duration estimated using the LOCA2‐hybrid were about 0.3 weeks longer on average than those from dynamically downscaled data by end of century, with smaller inland changes using dynamical downscaling (Figure 6).

## 4 Discussion

Our analysis reveals that differences in climate projection estimates across downscaled products can have important implications for projecting the health impacts of climate change. The LOCA2‐hybrid downscaled data indicate a more pronounced lengthening of the dry season, along with a sharper transition between wet and dry seasons compared to dynamically downscaled data. These differences have important implications for projecting future changes in coccidioidomycosis seasonal dynamics, in part because coccidioidomycosis seasonality and incidence is highly dependent on precipitation patterns (Comrie, 2005; M. E. Gorris et al., 2018; Head et al., 2022; Weaver & Kolivras, 2018). We project that the coccidioidomycosis transmission season will start earlier, end later, and generally extend longer in the future compared to historical patterns. The longer dry seasons and more intense wet‐dry season transitions predicted by LOCA2‐hybrid yield greater changes in coccidioidomycosis seasonality relative to estimates generated using the dynamically downscaled data. Coccidioidomycosis seasonality projections using different downscaling methods also exhibited key spatial differences that could have implications for policy and practice. For example, the end of the coccidioidomycosis transmission season in the Central Valley by end of the century projected using the LOCA2‐hybrid product may prompt extended targeted exposure reduction efforts when compared with projections using dynamically downscaled data. Similarly, when estimated using the LOCA2‐hybrid product, transmission season duration estimates are projected to be approximately ∼1–2 weeks longer by end of century across the Central Valley relative to dynamical downscaling. Thus, choice of downscaled climate data set could potentially impact decisions regarding exposure reduction measures for outdoor agricultural workers as the transmission season shifts to overlap with different periods during the growing, harvesting, and pre‐planting seasons. In the Central Valley, peak planting and harvesting activities typically occur from late spring through the fall, with crops like grapes, tomatoes, nuts, and lettuce requiring significant outdoor labor during these months. If the transmission season begins earlier or lasts longer, workers may be exposed to higher risk during critical agricultural periods when outdoor activity cannot easily be postponed, underscoring the importance of using reliable climate‐health projections to guide workplace safety and mitigation strategies.

Our study has multiple limitations, including uncertainties introduced by differences in spatial resolution between the downscaled data products (9 km for dynamically downscaled data vs. 3 km for the LOCA2‐hybrid). Additionally, our analysis is limited to a small number of GCMs and a single SSP. While a broader range of GCMs and SSPs would enhance our understanding of future coccidioidomycosis seasonal dynamics in California, focusing on the same three GCMs across both downscaling methods allows us to isolate the impact of downscaling technique on public health projections. Future work should expand the number of GCMs and SSPs used in order to better understand projected changes in coccidioidomycosis seasonal dynamics under a range of possible future scenarios. Finally, we utilize temperature and precipitation as proxies for soil moisture, which is likely more directly associated with Coccidioides growth and initiation of airborne dispersion (Coopersmith et al., 2017). While temperature, precipitation, and soil moisture are highly correlated historically, these relationships may weaken in the future as climate change progresses in California (Statewide Summary Report, 2018).

Differences between statistical and dynamical downscaling outputs themselves and the climate impacts they project can arise from several sources, including the fundamental methods themselves, the native resolution of the driving GCMs, the resolution of the downscaled output, bias‐correction strategies, and the observational or training data sets applied. Given these multiple interacting factors, no single driver may explain all of the differences across downscaling methods. While accounting for all of these factors is likely outside of the scope of most climate‐related health studies and other efforts to quantify the downstream impacts of climate change, they should be acknowledged when interpreting the results of climate impact analyses.

Determining which climate projections most realistically represent the future is challenging, as future projections are inherently uncertain. Additionally, historical comparisons among downscaled climate products do not necessarily reflect the validity of future projections. These inconsistencies may reflect fundamental differences in downscaling methodologies, that is, dynamically downscaled models can respond to changing climate conditions, whereas statistical models like LOCA2‐hybrid cannot. In the absence of definitive validation, which is impossible when projecting into the future, one approach is to conduct health impact analyses using both downscaling methods, if available for the region of interest, in order to consider a range of possible outcomes. Another approach would be to hypothesize the specific local climate mechanisms that will drive changes in your health outcome of interest and to engage with climate scientists who produce these data sets to better understand uncertainties and consider implications for health impact projections. While there are not necessarily quantitative ways to validate future projections, climate scientists may be able to provide expert opinions on which data set may be more appropriate, given their knowledge of local and regional climate processes that may be better captured using one method over the other (e.g., precipitation changes in the Sierra Nevada). Further, climate projections downscaled using multiple methods may not be available for specific regions of interest, particularly dynamically downscaled data given the computational expense. So, while it is important that climate scientists continue to develop these data sets for use in impact analyses, in the absence of multiple options, it is still important for public health researchers to understand how uncertainties in their chosen climate product may impact their health impact projections. We provide a list of key questions and considerations for public health researchers looking to incorporate climate projections into their analyses in Box 1.

**Box 1 Key questions and considerations for selecting downscaled climate data products for future health impact studies**

1 If the answer to any of these questions is “yes,” the choice of downscaling method could significantly affect your results. To better capture the range of potential health impacts, consider using multiple downscaling approaches, if available. Some of these questions may fall outside the typical expertise of public health researchers. In such cases, collaboration with climate scientists is strongly encouraged.

1. Is the health outcome of interest sensitive to intra‐annual climate patterns?
2. Does the region of interest contain complex terrain or localized climate patterns that cause sharp gradients in your climate variables of interest?
3. Does the health outcome depend more strongly on climate variables that are less predictable across data sets (e.g., precipitation) compared to those that are generally more robust (e.g., temperature)?
4. Do downscaled historical projections align well with observed data in the region of interest?
5. Do climate variable biases differ by season or region?
6. Are future projections more variable across methods than historical simulations?
7. Does the magnitude of that variability change during your future time period?

The sensitivity of health impact projections to choice of climate data downscaling method raises broader questions about other climate‐related exposures. Wildfire risk, which can also depend on the wet‐dry season transition, may be similarly influenced by differences across downscaled climate products (Abatzoglou et al., 2018; Guirguis et al., 2023; Swain, 2021). Other climate‐related exposures such as extreme weather events, flood risk, and vector‐borne disease risks could also be sensitive to choice of climate data source. While not all health burden projections will be impacted by differences across climate data sets (e.g., temperature projections in our study were relatively consistent across methods), we have demonstrated that some climate‐related health impacts are indeed sensitive to these differences. This may be particularly true for outcomes that are closely tied to complex, sub‐annual climate patterns, necessitating careful consideration in future climate‐related health impact studies.

## Conflict of Interest

The authors declare no conflicts of interest relevant to this study.

## Supporting information

Supporting Information S1

## Data Availability

Dynamically downscaled (https://wrf‐cmip6‐noversioning.s3.amazonaws.com/index.html) and LOCA2‐hybrid (https://cadcat.s3.amazonaws.com/index.html#loca2/) climate data were retrieved from publicly available AWS S3 repositories. PRISM climate data are publicly available from the PRISM Climate Group at Oregon State University (PRISM Group, 2025). PRISM data were accessed using the “prism” R package (https://rdocumentation.org/packages/prism/versions/0.1.0).
