# Supplementary material for: Choice of Downscaled Climate Product Matters: Projections of Valley Fever Seasonality in a Warming Climate
Source: Geohealth. 2025 Dec 31;10(1):e2025GH001624. doi: 10.1029/2025GH001624 (PMC12754269; doi:10.1029/2025GH001624)
Supplement: Supplementary file 1 — Supporting Information S1 [file GH2-10-e2025GH001624-s001.docx]

**Downscaling Method Matters: Projections of Valley Fever Seasonality in a Warming Climate**

**Authors:** Claire L. Schollaert^1*^, Simon Camponuri^2^, Lisa Couper^2^, Jennifer R. Head^3,4^, Alexandra Heaney^5^, Stefan Rahimi^6^, Justin V. Remais^2^, Miriam E. Marlier^1^

**Affiliations**

^1^ 1 Department of Environmental Health Sciences, UCLA Fielding School of Public Health, Los Angeles, CA, USA

^2^ Division of Environmental Health Sciences, School of Public Health, University of California, Berkeley, Berkeley, CA, USA

^3^ Department of Epidemiology, School of Public Health, University of Michigan, Ann Arbor, MI, USA

^4^ Institute for Global Change Biology, University of Michigan, Ann Arbor, MI 48109

^5^ Herbert Wertheim School of Public Health and Human Longevity, University of California San Diego, San Diego, CA, USA

^6^ Department of Atmospheric Science, University of Wyoming, Laramie, WY, 82071 USA

*corresponding author: Claire Schollaert (cschollaert@ucla.edu)

**Table of Contents:**

Table S1

Table S2

Figure S1

Figure S2

Figure S3

Figure S4

Figure S5

**Table S1.** Annual and seasonal correlation coefficients between weekly census-tract level historical temperature and precipitation estimates from the two downscaling approaches and the PRISM reference data.

|  | **Season** | **LOCA2-hybrid vs**  **Dynamical** | **LOCA2-hybrid vs**  **PRISM** | **Dynamical vs**  **PRISM** |
| --- | --- | --- | --- | --- |
| Temperature | Annual | 0.99 | 0.99 | 0.98 |
|  | Spring | 0.96 | 0.97 | 0.93 |
|  | Summer | 0.95 | 0.97 | 0.93 |
|  | Fall | 0.98 | 0.99 | 0.97 |
|  | Winter | 0.94 | 0.97 | 0.92 |
| Precipitation | Annual | 0.96 | 0.98 | 0.95 |
|  | Spring | 0.96 | 0.97 | 0.97 |
|  | Summer | 0.33 | 0.63 | 0.00 |
|  | Fall | 0.96 | 0.97 | 0.97 |
|  | Winter | 0.87 | 0.95 | 0.85 |

Table S2. Variability in monthly average temperature and precipitation estimates across downscaling methods (e.g. spread across LOCA2-Hybrid and dynamically downscaled CNRM-ESM2-1 estimates) and across GCMs (e.g. spread across GCMs under each downscaling method).

|  | **Temperature** | | | **Precipitation** | | |
| --- | --- | --- | --- | --- | --- | --- |
|  | **Range (°C)** | **SD (°C)** | **IQR (°C)** | **Range (mm/wk)** | **SD (mm/wk)** | **IQR (mm/wk)** |
| **Spread across downscaling methods** | | | | | | |
| CNRM-ESM2-1 | 1.4 | 1 | 0.7 | 2.3 | 1.6 | 1.2 |
| EC-Earth3-Veg | 1.1 | 0.8 | 0.6 | 1.3 | 0.9 | 0.7 |
| FGOALS-g3 | 1.7 | 1.2 | 0.9 | 3.3 | 2.3 | 1.6 |
| **Spread across GCMs** | | | | | | |
| LOCA2-Hybrid | 0.5 | 0.2 | 0.2 | 1.8 | 1 | 0.5 |
| Dynamical | 0.3 | 0.1 | 0.2 | 1.0 | 0.5 | 0.3 |


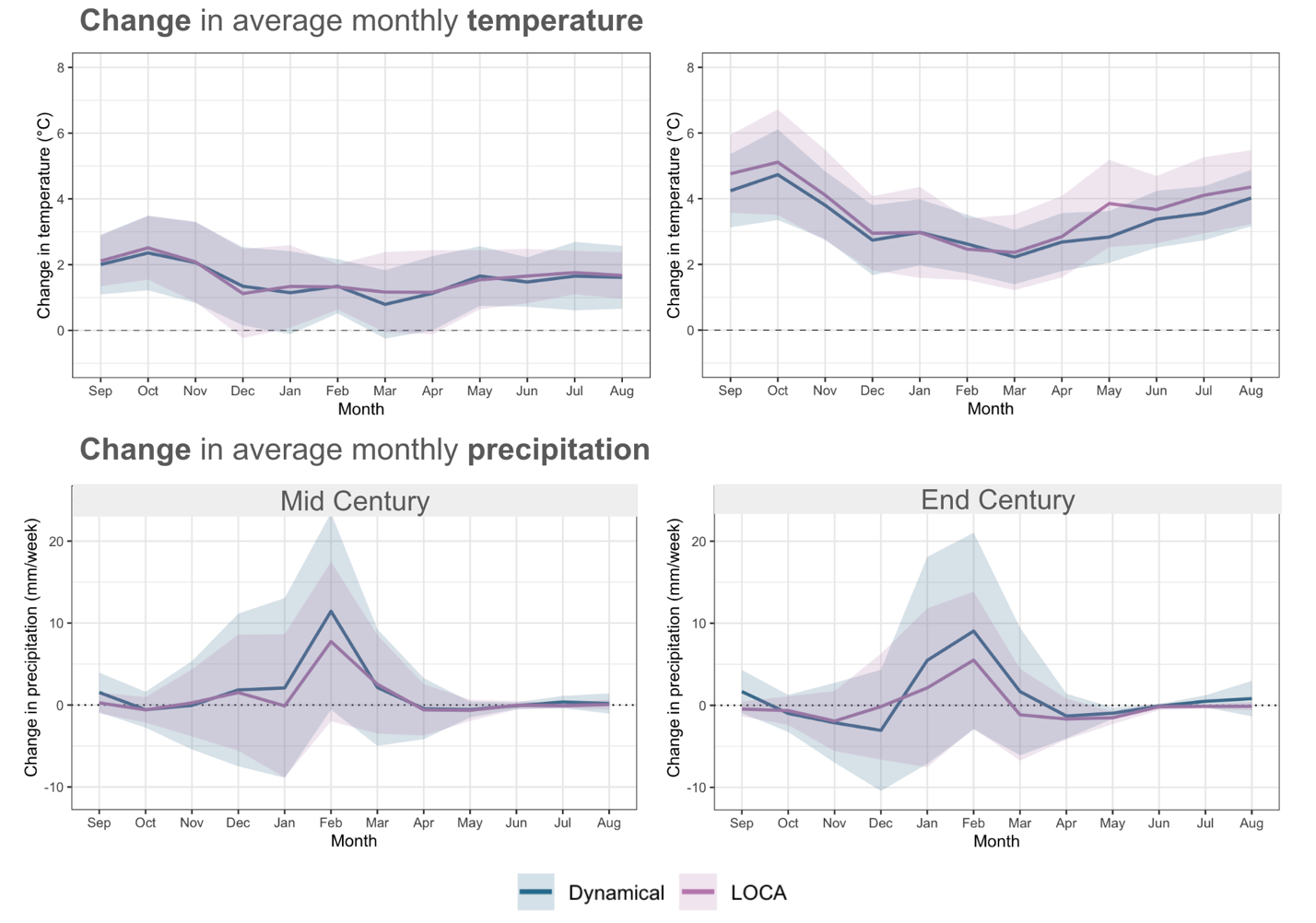


**Figure S1.** Study region census-tract level average temperature (top row) and precipitation (bottom row) estimates across historical (1980-2010) period derived using PRISM data. The middle and right panels show relative difference in average temperature and precipitation compared to PRISM under each downscaling method.


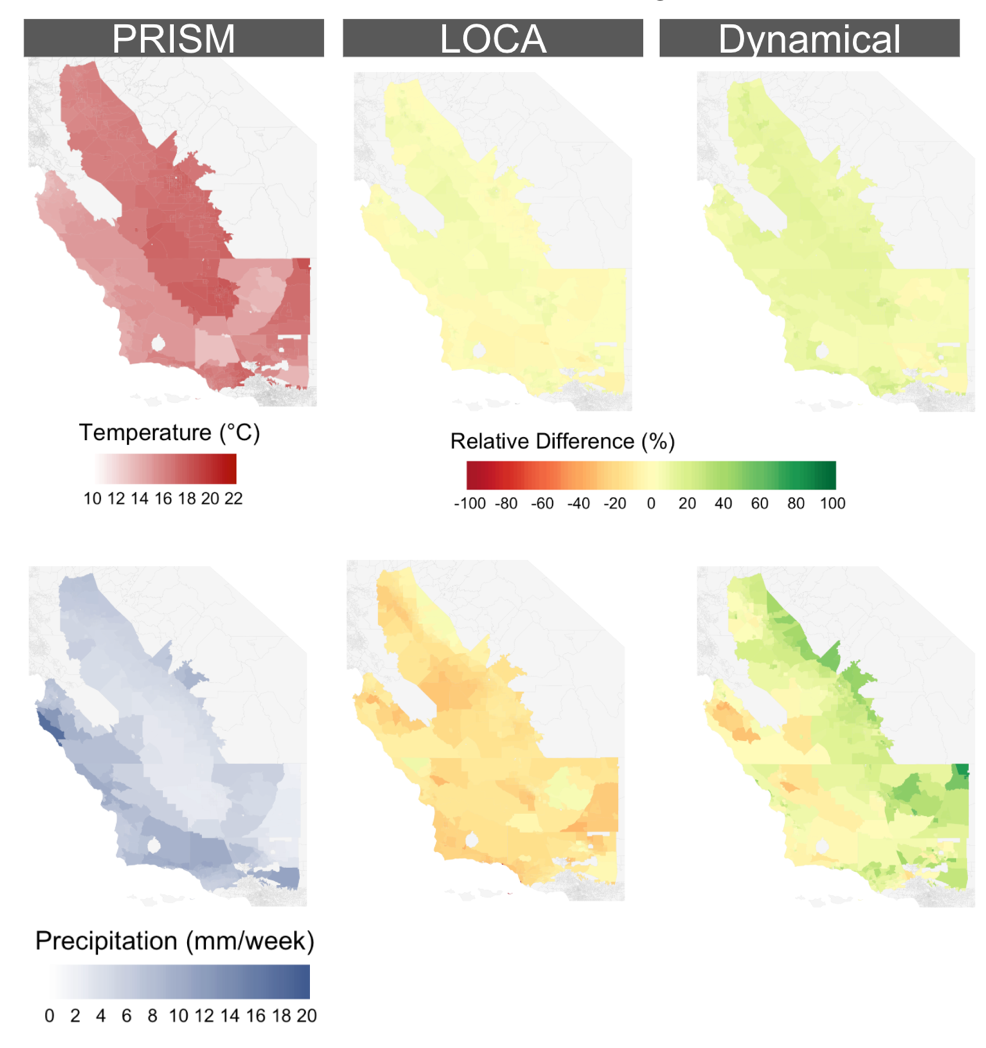


**Figure S2.** Change in average monthly temperature (top) and precipitation (bottom) by mid and end of century across downscaling methods relative to the historical period.


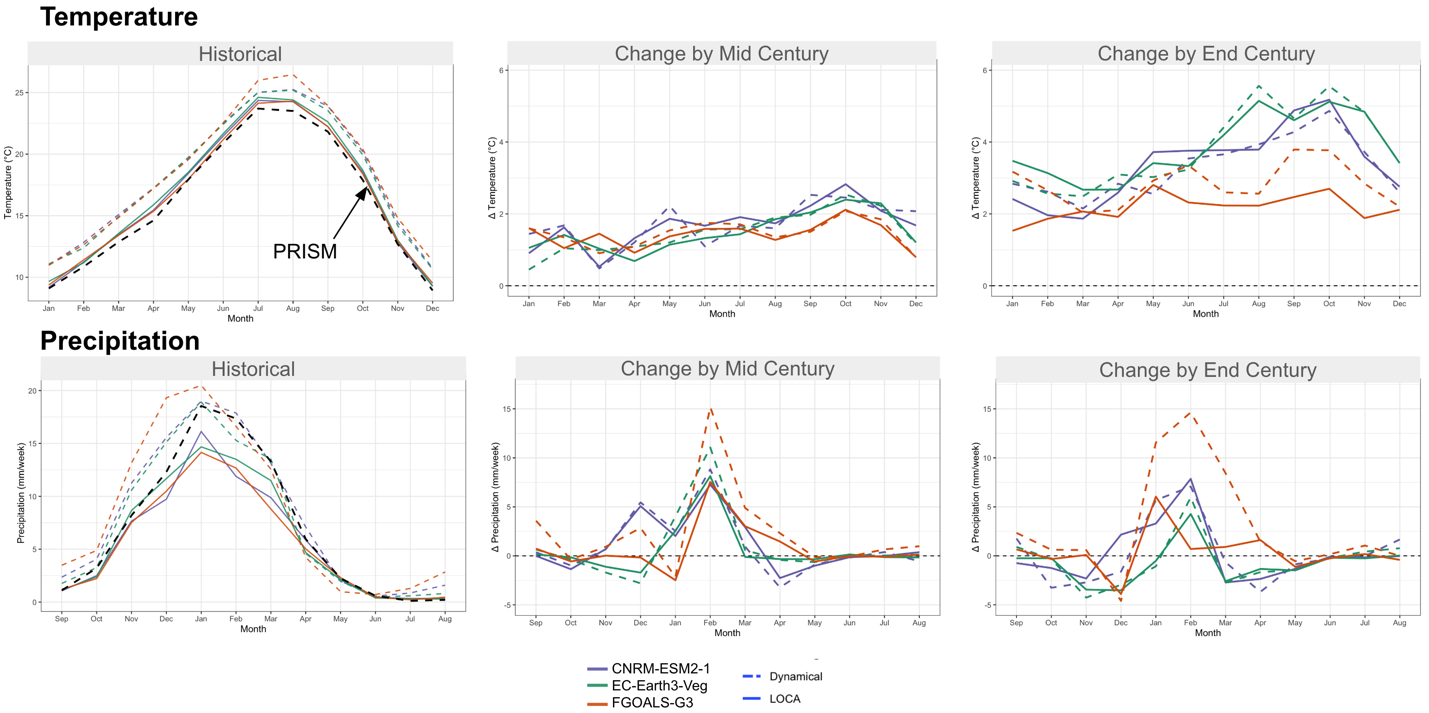


**Figure S3**. Historical average monthly temperature (top) and precipitation (bottom) and change relative to historical by mid and end of century across GCMs and downscaling methods.


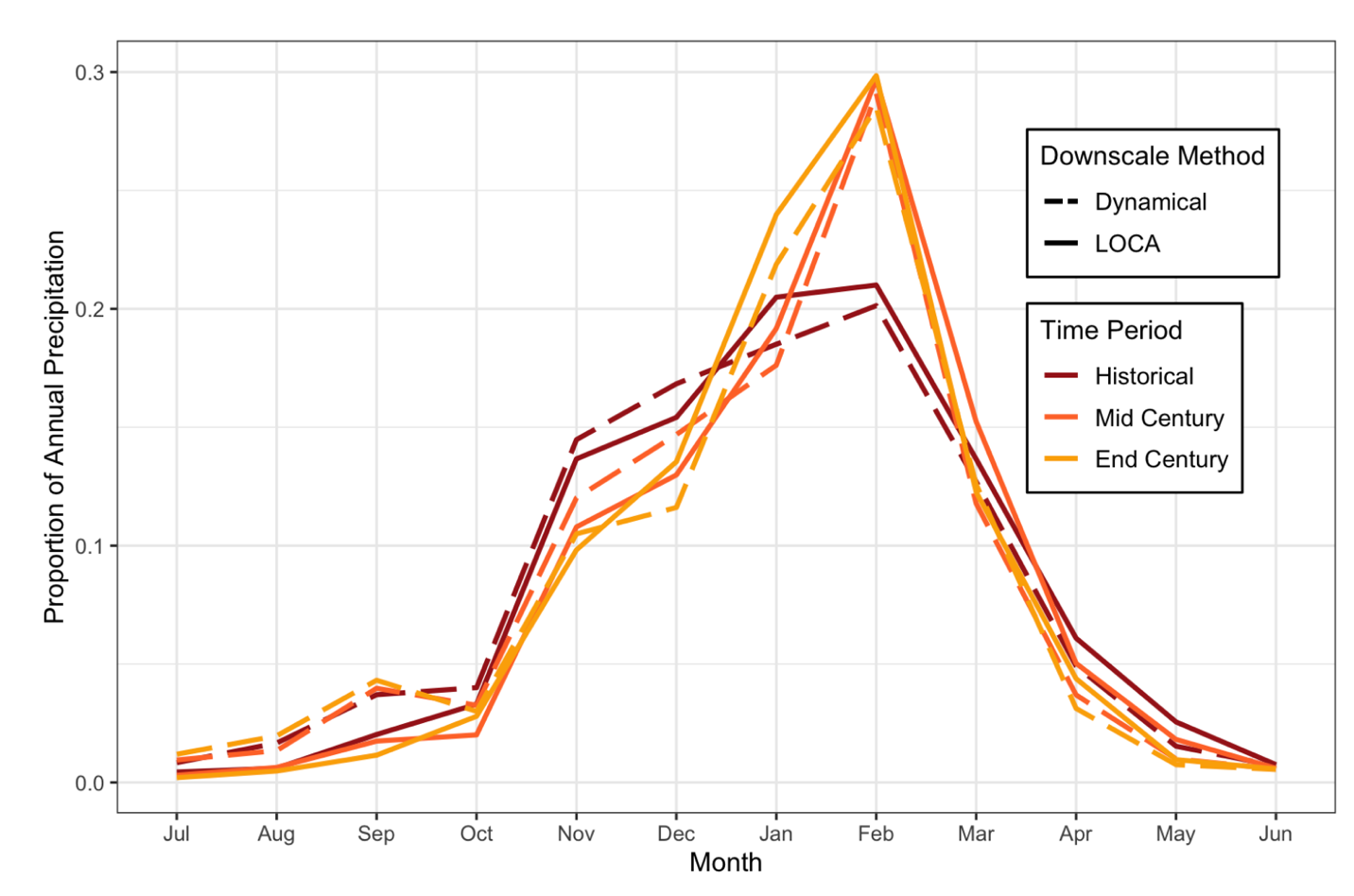


**Figure S4.** Proportion of total annual precipitation that falls within each month across each time period and downscaling method.

**
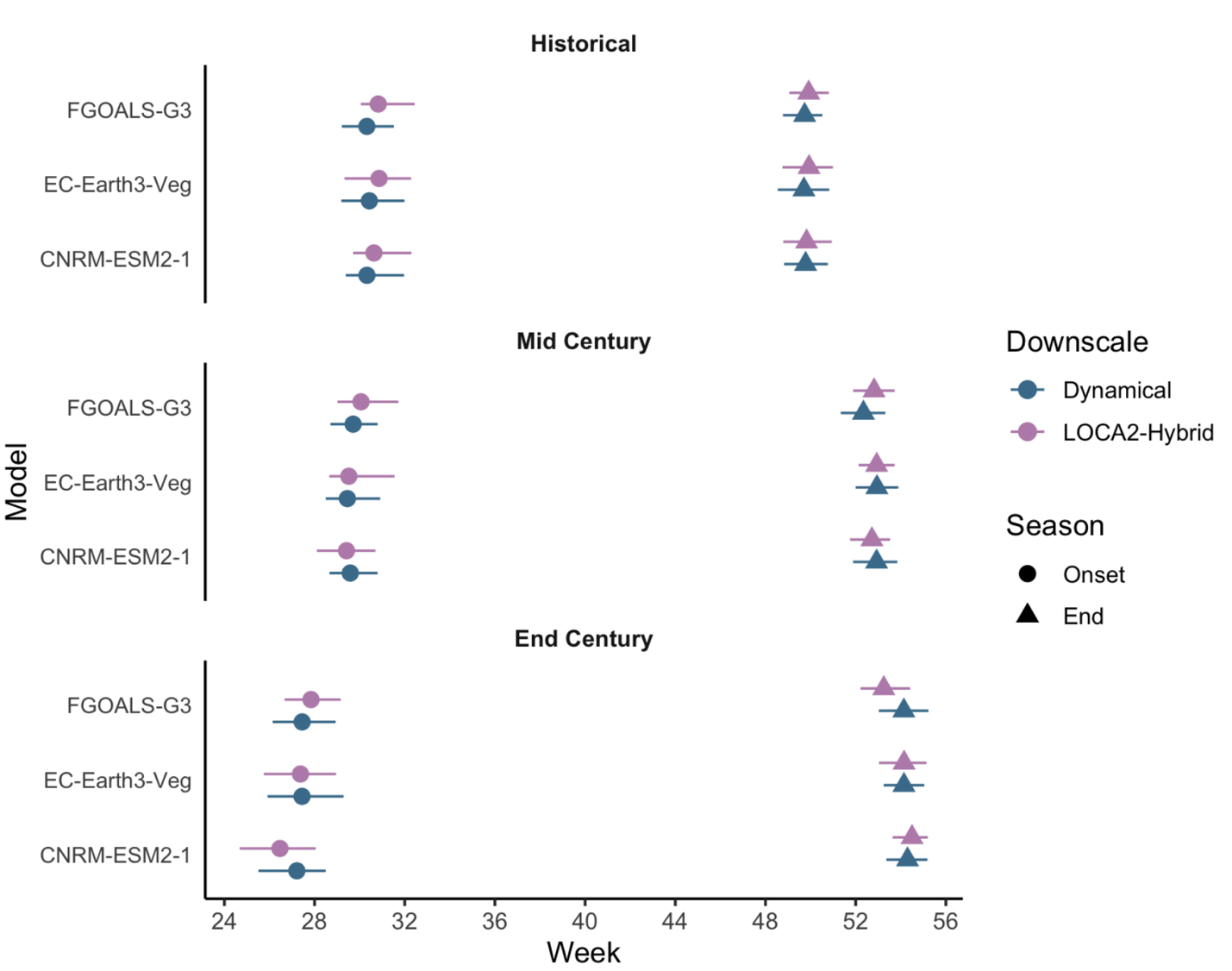
**

**Figure S5**. Median and IQR of season onset and end weeks over the course of the century estimated using temperature and precipitation projections from both downscaling methods and each GCM individually. Bars represent the spread across census tracts and years within each time period.
